# Report of Seventh District Ohio Dental Society

**Published:** 1889-12

**Authors:** E. G Betty

**Affiliations:** Cincinnati


### Report of Seventh District, Ohio Dental Society.

BY E. G BETTY, D D.S., CINCINNATI.

Read before the Ohio State Dental Society at Cleveland, October, 1889.

In making this report to the State body I considered it my duty, in view of the fact that the districts are all subordinate to the State Society, and that it therefore is entitled to a knowledge of what has been done by them, I shall, therefore, briefly review the work accomplished by the Seventh District.

The preliminary meeting, pursuant to call by printed notices, was held in Dayton, Montgomery county, May 21st of the current year, at the Phillips House. Thirteen professional brothers responded to the call and took active part in the work of organization, the attendance representing Hamilton, Montgomery, Butler, Greene, Preble and Clinton counties, being a little over half of the counties included in our district.

The object of the meeting was announced by State-committeeman, Dr. J. Taft, all present engaging in the discussion. All expressed themselves as favorable to the plan, and on motion, adopted the constitution, by-laws, and order of business suggested by the State committee.

The election of officers for the year resulted in the choice of E. G. Betty, Hamilton county, President; L. E Custer, Montgomery county, Vice-President; Chas. Welch, Clinton county, Secretary; Chas. I. Keely, Butler county, Treasurer.

J. Taft, Hamilton county, W. H. Sillito, Greene county, L. C. Adams, Montgomery county, Executive Committee.

The society then adjourned to re-assemble on the third Tuesday of September (17th inst), 1889.

Upon that day the society met in Lincoln Club Hall, Cincinnati, a good attendance present. Interesting papers were read and discussed to the satisfaction and benefit of the members.

Dr. C. M. Wright's paper propounded the question, "Has a Dentist the Legal or Moral Right to Practice General Medicine."

Dr. J. Taft read a paper on "Clinical Instruction."

Dr. J. R. Callahan's paper treated of "The Care of Children's Teeth."

All these papers will appear in print, so that many others may enjoy and profit by them. In addition to the papers and discussions, a number of interesting cases, specimens, models and appliances were exhibited and commented upon.

It is our intention to hold clinics hereafter; a wise conclusion.

During the meeting nine new members, all from Hamilton county, were elected, the roster now showing a total membership of twenty-two (22), a splendid record for so young a society.

The chairman in his opening address suggested the propriety and feasibility of the District Societies making a complete roster of the dentists of the State of Ohio. By resolution, the Executive Committee, consisting of Drs. J. Taft, H. A. Smith, and L. E. Custer, was instructed to list the dentists practicing in the Seventh District.

I mention this matter at this time, Mr. Chairman, in order to bring it before the State Society that it may assist the several districts in consummating this important work.

In conclusion, I would like to suggest that the presentation of annual reports from the districts be given a permanent place on the program of the annual meetings of the State Society, in order that the parent body may have a complete knowledge of what is being done throughout the State and inure to the benefit of all.

---

If asked what, as the result of my experience, is the greatest pleasure of my life, I should say, doing good to others. Not a strikingly original remark, perhaps, but seemingly the most difficult thing in the world is to be prosperous and generous at the same time.—*Geo. W. Childs.*

